# Whole-Body Acute Contact Toxicity of Formulated Insecticide Mixtures to Blue Orchard Bees (*Osmia lignaria*)

**DOI:** 10.3390/toxics9030061

**Published:** 2021-03-17

**Authors:** Joseph Belsky, David J. Biddinger, Neelendra K. Joshi

**Affiliations:** 1 Department of Entomology and Plant Pathology, University of Arkansas, Fayetteville, AR 72701, USA; jebuark@gmail.com; 2 Department of Entomology, The Pennsylvania State University, University Park, PA 16801, USA; djb134@psu.edu; 3 Penn State Fruit Research and Extension Center, Biglerville, PA 17307, USA

**Keywords:** whole-body contact toxicity, neonicotinoids, pyrethroids, combined active ingredients, non-*Apis* bees, pollinators

## Abstract

Blue orchard bees, [*Osmia lignaria* (Say) (Hymenoptera: Megachilidae)], have been developed as an important pollinator for orchard crops in North America over the last 40 years. The toxicity of several pesticides to *O. lignaria* and other *Osmia* species has been previously reported. However, the field-realistic toxicity of formulated premix insecticides comprised of multiple active ingredients (each with a different mode of action) to *O. lignaria* has not been assessed. Here, we use a customized spray tower in a laboratory setting to assess adult male and female whole-body direct contact exposure to four formulated pesticide mixtures: thiamethoxam + lambda-cyhalothrin (TLC), imidacloprid + beta-cyfluthrin (IBC), chlorantraniliprole + lambda-cyhalothrin (CLC) and methoxyfenozide + spinetoram (MS) by directly spraying anesthetized bees in Petri dishes. Separately, adult male and female whole-body direct contact exposure to formulated imidacloprid (I), beta-cyfluthrin (BC) and their 1:1 binary combination (IBC) was assessed using the same experimental method. Resulting mortality in each study was screened up to 96 h post-treatment to determine acute whole-body contact toxicity. In the first study, TLC and IBC resulted in statistically higher mortality at 24 and 48 h than the two other insecticide combinations tested. The CLC and MS combinations were slower acting and the highest mortality for *O. lignaria* exposed to these mixtures was recorded at 96 h. We did observe significant differences in toxicity between CLC and MS. In the second study, exposure to the 1:1 binary combination of IBC caused overall significantly higher mortality than exposure to I or BC alone. Both active ingredients alone, however, demonstrated equivalent levels of mortality to the 1:1 binary combination treatment at the 96 h observation reading, indicating increased speed of kill, but not necessarily increased toxicity. Significant differences in the onset of mortality following acute contact whole-body exposure to the formulated insecticide mixtures and individual active ingredients tested were consistently observed across all experiments in both studies.

## 1. Introduction

Blue orchard bees, [*Osmia lignaria* (Say) (Hymenoptera: Megachilidae)] are solitary nesting bees native to North America that are increasingly being utilized for their pollination services in commercial crop production [1,2,3,4]. Like other bee species, *O. lignaria* are also exposed to multiple environmental stressors impacting their reproductive success, overall population decline and ultimately the ability to provide effective pollination services [5]. One potential environmental stressor of interest is assessing the effects of pollinator exposure to pesticides commonly used in orchards before and after bloom. Ample research has been done on honey bees, [*Apis mellifera* (Linnaeus) (Hymenoptera: Apidae)] and more recently, on alternative pollinators such as *O. lignaria* and other related *Osmia* species. Although pesticide applications in orchards are restricted during bloom, pollinators can still be exposed to systemic insecticide residues present in flowers from pre-bloom sprays [6]. Additionally, insecticide applications made before the bloom is complete (as a result of various percentages of petal fall) can also result in pollinator exposure. In the case of *A. mellifera*, hives can be moved to safe locations, but for *Osmia* and other wild pollinators whose nests cannot be moved once initiated, this poses a greater problem. Moreover, while *A. mellifera* hives can be thought of as a super-organism with thousands of specialized workers, drones (males) and an egg-laying queen that is protected, in *Osmia* spp. and other solitary bees there is only an egg-laying female that upon being fertilized by a male, serves as a worker and forager for food, and constructs nests from plants or mud where they can also be exposed to pesticides by different routes [1,5]. Losing a few workers in hives of 10,000 individuals or more from pesticides can be compensated for with *A. mellifera*, which reproduce in large numbers and have multiple generations a year, but with *Osmia* spp. the loss of any female individual is the loss of a reproductive and leads to the loss of the next generation, which is especially devastating since most *Osmia* spp. are univoltine [1,5]. Males are equally important in *Osmia* spp. as they are responsible for fertilizing females and also provide pollination services.

Compared to the literature for *Apis* spp. (honey bees and bumble bees), relatively few studies have evaluated pesticide toxicity to *O. lignaria* and only for a few compounds. For instance, dosing *O. lignaria* larval provisions with the neonicotinoid imidacloprid resulted in prolonged development and cocoon spinning times for larvae exposed to the higher doses, however no lethal effects were detected [7]. A study evaluating contact and oral toxicity of five fungicides to *O. lignaria* found acute and delayed oral toxicity from single high doses of the fungicide propiconazole and high acute and delayed oral and contact toxicity from the fungicide captan [8]. Pesticide exposure also has been found to impact *O. lignaria* behavior. For example, one study analyzed the effects of sprayed pesticide exposure on almond trees within a cage enclosure in a field setting [9]. This study demonstrated that the fungicides iprodione, pyraclostrobin + boscalid and the non-ionic wetting agent N-90^®^ (90% polyethoxylated nonylphenol) inhibited *O. lignaria* female nest recognition.

The development of resistance to existing insecticide chemistries has dramatically increased in several arthropod pest species in recent years [10,11]. In response, several chemical companies have developed premix insecticides comprised of multiple active ingredients (each with a different mode of action) aimed at combatting insecticide resistance development. The toxicology of these formulated premix insecticides (some of which are neonicotinoids) to solitary bees in general and the blue orchard bee in particular has not been tested. Studies investigating contact exposure of premix insecticides to honey bees have demonstrated high toxicity in terms of low lethal doses that kill 50% of the test subjects (LD_50_) values in mortality screenings up to 48 h after treatment [12,13,14]. We expand upon the findings of these studies in *A. mellifera* by simulating adult male and female *O. lignaria* direct contact exposure to four premix insecticides used in tree fruit. Commercially managed *A. mellifera* live in portable hives that can be moved into pollination events and removed prior to application of insecticides. Conversely, managed solitary bees (including *O. lignaria*) live in nests that cannot be removed before an insecticide application due to nesting behavior [1,5]. Additionally, since they cannot be removed at 80% petal fall which is a common timing for post-bloom pesticide applications, *Osmia* spp. are at a higher risk for insecticide exposure during the process of foraging. Moreover, since each female is a reproductive, female success is directly linked to offspring success [1].

Here we examine male and female blue orchard bee onset of mortality following insecticide exposure under the application scenario of being contacted by air-blast sprays while foraging through an orchard during late bloom. In our first study, we specifically assessed differences in the onset of mortality following whole-body contact exposure to the different premix insecticide combinations over an extended screening of 24, 48, 72 and 96 h post-treatment (Experiments 1 and 2). In our second study (for the imidacloprid + beta-cyfluthrin premix), we assess differences in the onset of mortality following whole-body contact exposure to each active ingredient alone and mixed together in a 1:1 binary combination made in the laboratory using the same methodology (Experiment 3). These findings will aid orchard and field crop growers, private crop consultants and cooperative extension in the selection and use of insecticides that will minimize the impact on *O. lignaria* and other solitary bee pollinators that are both commercially managed and naturally occurring in agro-ecosystems.

## 2. Materials and Methods

### 2.1. General Experimental Design

#### 2.1.1. Bees

Cocoons containing pupae with adult male and female blue orchard bees in diapause were purchased from Watts Solitary Bees (Bothell, WA United States) during spring 2018. Cocoons were placed in an incubator at (5 ± 0.5 °C; 65 ± 5% RH) within a laboratory setting until they were used for experiments, which began three-to-seven weeks afterwards. Four days prior to beginning each experiment, cocoons were placed in an incubator with a higher temperature (14 ± 0.5 °C; 65 ± 5% RH) to stimulate adult pupal eclosion. *O. lignaria* used for all experiments had eclosed from their pupae less than 24 h prior to being treated.

#### 2.1.2. Experimental Cage Design

Transparent polypropylene jars (500 mL, D by H: 9.3 by 10.0 cm) were used as experimental cages. Fine netting cloth was securely placed with rubber bands over the jar opening to prevent escape. *O. lignaria* were immobilized by cooling in separate storage jars for approximately two minutes prior to being used in an experiment. Immobilized blue orchard bees were quickly transferred to Petri dishes where they were directly sprayed with their respective insecticide treatment inside the spray tower, and then placed in clean jars for the duration of each experiment. Treated *O. lignaria* were fed by inserting a plastic vial containing 50% sucrose solution (6–7 mL per vial) into the side of each cage for *ad libitum* feeding. A cotton swab soaked in 50% sucrose solution was placed on top of each cage providing a second food resource.

#### 2.1.3. Spray Tower

Insecticides were administered using a modified version of the spray tower design as described in Zhu et al. [12] to simulate direct foliar spray field exposure under more precise controlled conditions. A spray nozzle (Burkard Scientific, Uxbridge, United Kingdom) was used for applying insecticides to *O. lignaria* in cages at a low spray volume (0.5 mL/cage) in distilled water. Insecticide applications simulated mixture in 378.541 L of distilled water per hectare in an air-blast sprayer, and were scaled to the surface area of the spray tower. Insecticides were mixed in 100 mL of distilled water and applied with regulated air pressure (69 kpa) and fixed spray distance (22 cm) for each treatment including the distilled water control [12].

#### 2.1.4. Post Treatment Observations

Treated *O. lignaria* were transferred to clean jars placed on plastic trays, where they were maintained in an incubator at 16 h daylight and 8 h darkness (21 ± 0.5 °C; 65 ± 5% RH) for the duration of the experiment. Resulting mortality was recorded at 24, 48, 72, and 96 h after treatment for all treatments, except Experiment 3, which was terminated after 48 h for males only. *O. lignaria* were considered dead if they did not move after being touched by a paintbrush. Moribund bees capable of moving their legs and antennae but incapable of flying were counted as being alive.

### 2.2. Experiment 1: Male O. lignaria Acute Whole-Body Contact Toxicity to Premix Insecticides

Four formulated insecticide mixtures (Table 1) were assessed for their contact toxicity to adult male *O. lignaria* by directly spraying anesthetized bees in Petri dishes placed in the spray tower. Each of these insecticides is comprised of two active ingredients (each with a different mode of action). A single concentration for each insecticide was chosen that was in the middle of the concentration range of rates listed on the Environmental Protection Agency (EPA) regulated insecticide label for orchard crops (Table 2). We mixed each insecticide solute in 100 mL of a distilled water solvent. The control treatment was sprayed with distilled water at the same spray volume. For all treatments, we filled the plastic vial feeder inserted in each cage with an additional 2 mL of 50% sucrose solution at both 48 and 72 h for continued male *O. lignaria* feeding. A completely randomized design was used to test the four treatments and control in this experiment. Seven cages containing 10 male *O. lignaria* each (*n* = 10/cage, total 70 bees) were utilized for each of the five treatments, with each individual cage representing an experimental unit. Bees were randomly assigned to treatment groups and placed in cages representing an experimental unit.

### 2.3. Experiment 2: Female O. lignaria Acute Whole-Body Contact Toxicity to Premix Insecticides

Whole-body contact toxicity of four formulated premix insecticides (Table 1) to adult female *O. lignaria* was assessed by applying insecticides to bees following the same procedure as described in Experiment 1. A completely randomized design was used to test the four treatments and control in this experiment. Six cages containing nine female *O. lignaria* each (*n* = 9/cage, total 54 bees) were utilized for each of the four treatments and control. Each individual cage represented an experimental unit. Bees were randomly assigned to treatment groups and placed in cages representing an experimental unit.

### 2.4. Experiment 3: Male and Female O. lignaria Acute Whole-Body Contact Toxicity to Individual Active Ingredient Insecticides and 1:1 Binary Combinations

Whole-body contact toxicity of the formulated individual active ingredients (imidacloprid and beta-cyfluthrin) and their 1:1 binary combination (Table 3) to adult male and female *O. lignaria* was assessed following the same procedure as described in Experiments 1 and 2. We used a 1:1 binary combination of the two individual active ingredients as opposed to simply testing the concentration of each active ingredient to specifically simulate the premix insecticide formulation used in Experiments 1 and 2. A concentration within the low-range of concentrations on the EPA regulated insecticide label recommendations for orchard crops (Table 4) was selected. A completely randomized design was used to test the three treatments and control in this experiment. Six cages containing 8 male *O. lignaria* each (*n* = 8/cage, total 48 bees) were utilized for each of the three treatments and control, with each individual cage representing an experimental unit. Bees were randomly assigned to treatment groups and placed in cages representing an experimental unit. The same experimental design was used for females.

### 2.5. Statistical Analysis

We corrected the control *O. lignaria* mortality for each experiment at each screening period by using Schneider-Orelli’s formula as in Bibbs et al. and Williams et al. [15,16]. This formula counts the number of dead individuals (which is in accordance with how we recorded our data during the observation period) as opposed to Abbott’s formula that counts the number of alive individuals over an observation period. Using Equation (1):

(1) $$\mathrm{Corrected}\;\%\;\mathrm{Mortality}=\left(\frac{\mathrm{Mortality}\;\%\;\mathrm{in}\;\mathrm{treatment}-\mathrm{Mortality}\;\%\;\mathrm{in}\;\mathrm{control}}{100-\mathrm{Mortality}\;\%\;\mathrm{in}\;\mathrm{control}}\right)*100$$

we compared the percent mortality in each experimental unit to the percent mortality in the respective control unit. This procedure was quantified for each of the four treatments in Experiments 1 and 2 and the three treatments in Experiment 3.

We removed the control mortality from each experiment as a result of correcting our data using Schneider–Orelli’s formula. Therefore, we only analyzed the *O. lignaria* mortality for insecticide treatments. An arcsine transformation was used to normalize corrected percent mortality data as in Glazer et al. [17]. Normal Q-Q and residual versus fitted plots were used to confirm normality and lack of heteroscedasticity.

Statistical significances in the percent of *O. lignaria* mortality following acute contact exposure to each of the four premix insecticides (Experiments 1 and 2) and to each individual active ingredient insecticide and their 1:1 binary combination (Experiment 3) tested at each screening period were determined using one-way ANOVA as in Wise et al. [18].

A Tukey’s HSD post hoc multiple pairwise comparison analysis was separately used to determine statistical differences in the percent of *O. lignaria* mortality following acute contact exposure to each premix insecticide (Experiments 1 and 2) and to each individual active ingredient insecticide and their 1:1 binary combination (Experiment 3) treatment during each screening period as in Wise et al. [18]. R statistical software (R version 3.5.1 and R Studio version 1.1.463) was used for completing all statistical analyses. The following libraries were utilized: datasets, emmeans, lsmeans and multcompView.

## 3. Results

### 3.1. Experiment 1: Male O. lignaria Acute Whole-Body Contact Toxicity Following Whole Bodily Contact Exposure to Premix Insecticide Sprays

**Figure 1:**
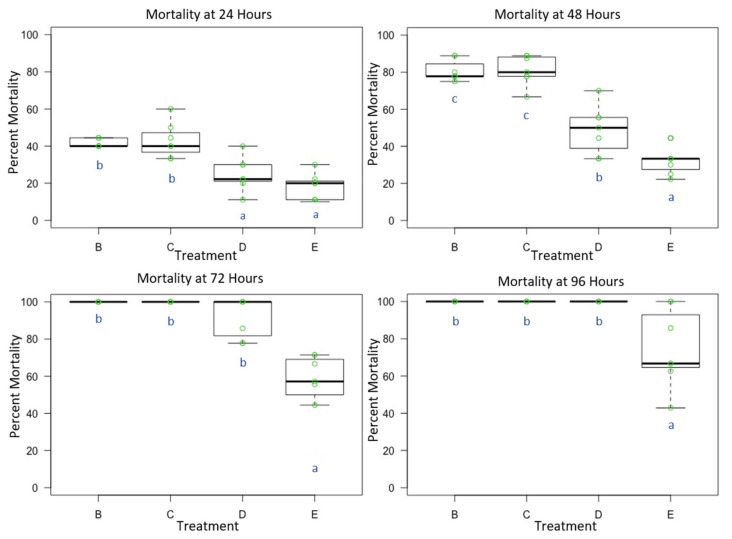
Male *Osmia lignaria* Acute Contact Toxicity Following Whole-Body Contact Exposure to Premix Insecticides: Male *Osmia lignaria* mean, 1st and 3rd quartile percent mortality following whole-body contact exposure to each insecticide mixture (Experiment 1) at 24, 48, 72 and 96 h. Data on the x-axis indicate each insecticide treatment, and data on the y-axis indicate percent mortality. Insecticide treatments: B = TLC, C = IBC, D = CLC, E = MS. All jars in a treatment containing the respective percent mortality are represented by a single green point. Letters (a, b, c) indicate significant difference at *p* < 0.05 (pairwise comparison). Information related to control mortality is given in the result section.

Male *O. lignaria* whole-body contact exposure to all four premix insecticide sprays (Table 1 and Table 2) resulted in mortality that was statistically significant between treatments at 24 h (F = 18.059, *p* < 0.05, df = 3), 48 h (F = 48.053, *p* < 0.05, df = 3), 72 h (F = 35.37, *p* < 0.05, df = 3) and 96 h (F = 12.824, *p* < 0.05, df = 3). At 24 h, thiamethoxam + lambda-cyhalothrin (TLC) and imidacloprid + beta-cyfluthrin (IBC) were more toxic than chlorantraniliprole + lambda-cyhalothrin (CLC) and methoxyfenozide + spinetoram (MS) (*p* < 0.05) (Figure 1). At 48 h, TLC and IBC were more toxic than CLC and MS, however, CLC was also statistically different from MS (*p* < 0.05). At 72 and 96 h, TLC, IBC and CLC were more toxic than MS (*p* < 0.05). At 24 h, average control mortality was 4.29% with a standard error of 2.02%. At 48 h, average control mortality was 10.0%, with a standard error of 2.18%. Cumulative mortality in each treatment further increased as time progressed, and at 72 h, average control mortality was 21.43%, with a standard error of 4.04%. At 96 h, average control mortality was 25.71%, with a standard error of 3.69%.

### 3.2. Experiment 2: Female O. lignaria Acute Contact Toxicity Following Whole-Body Contact Exposure to Premix Insecticides

**Figure 2:**
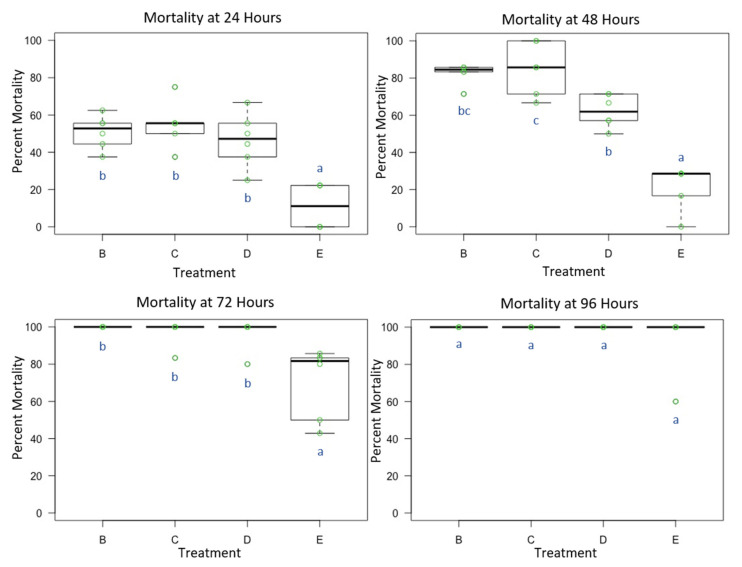
Female *Osmia lignaria* Acute Contact Toxicity Following Whole-Body Contact Exposure to Premix Insecticides: Female *Osmia lignaria* mean, 1st and 3rd quartile percent mortality following whole-body contact exposure to each insecticide mixture (Experiment 2) at 24, 48, 72 and 96 h. Data on the x-axis indicate each insecticide treatment, and data on the y-axis indicate percent mortality. Insecticide treatments: B = TLC, C = IBC, D = CLC, E = MS. All jars in a treatment containing the respective percent mortality are represented by a single green point. Letters (a, b, c) indicate significant difference at *p* < 0.05 (pairwise comparison). Information related to control mortality is given in the result section.

Female *O. lignaria* whole-body contact exposure to all four premix insecticide sprays (Table 1 and Table 2) resulted in female blue orchard bee mortality that was statistically significant between treatments at 24 h (F = 14.859, *p* < 0.05, df = 3), 48 h (F = 22.135, *p* < 0.05, df = 3) and 72 h (F = 14.869, *p* < 0.05, df = 3). At 96 h, we did not observe statistically significant mortality for all treatments (F = 1, *p* =0.413, df = 3) given the complete mortality at this screening period. However, one replication in MS was an outlier at 96 h with 60% mortality (Figure 2). At 24 and 72 h, TLC, IBC, and CLC were more toxic than MS (*p* < 0.05). At 48 h, TLC was more toxic than MS, while IBC was more toxic than CLC and MS, and CLC was more toxic than MS (*p* < 0.05). At 96 h, no differences in toxicity were observed between insecticide treatments given the observation of complete mortality (*p* > 0.05). Control mortality (for standard duration) at 24 h was 5.56% with a standard error of 2.48%, while it was relatively higher at 48 h (25.93% with a standard error of 2.34%) At 72 h, average control mortality was 31.48% with a standard error of 3.41%, and at 96 h, it was 46.30% with a standard error of 4.54%.

### 3.3. Experiment 3: Male and Female O. lignaria Acute Contact Toxicity Following Whole-Body Contact Exposure to Individual Active Ingredient Insecticides and 1:1 Binary Combinations

**Figure 3:**
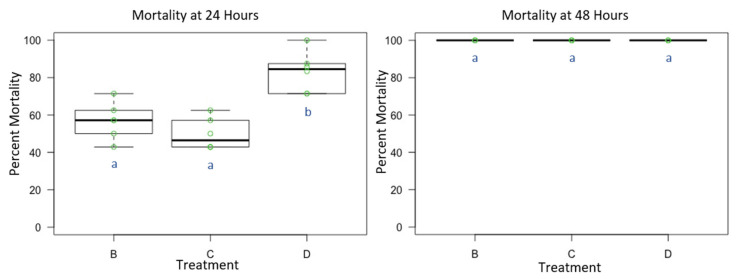
Male *Osmia lignaria* Acute Contact Toxicity Following Whole-Body Contact Exposure to Individual Active Ingredient Insecticides and their 1:1 Binary Combination: Male *Osmia lignaria* mean, 1st and 3rd quartile percent mortality following whole-body contact exposure to each individual active ingredient insecticide (Experiment 3) at 24 and 48 h. Data on the x-axis indicate each insecticide treatment, and data on the y-axis indicate percent mortality. Insecticide treatments: B = I, C = BC, D = IBC. All jars in a treatment containing the respective percent mortality are represented by a single green point. Significant difference at *p* < 0.05 (pairwise comparison) is indicated by different letters (a, b). Information related to control mortality is given in the result section.

Mortality for male *O. lignaria* following whole-body contact exposure to individual active ingredient insecticides and their 1:1 binary combination (Table 3 and Table 4) was statistically significant between treatments at 24 h (F = 13.098, *p* < 0.05, df = 2), however not at 48 h (F = 1, *p* =0.391, df = 2) given the complete mortality at this screening period which terminated the experiment early. The 1:1 binary combination (IBC) was more toxic than I or BC at 24 h (*p* < 0.05) (Figure 3). At 48 h, no difference in toxicity was observed between treatments given the complete mortality at this screening period (*p* > 0.05). At 24 h, average control mortality was 12.5% with a standard error of 3.23%. At 48 h, average control mortality was 18.75% with a standard error of 2.8%.

**Figure 4:**
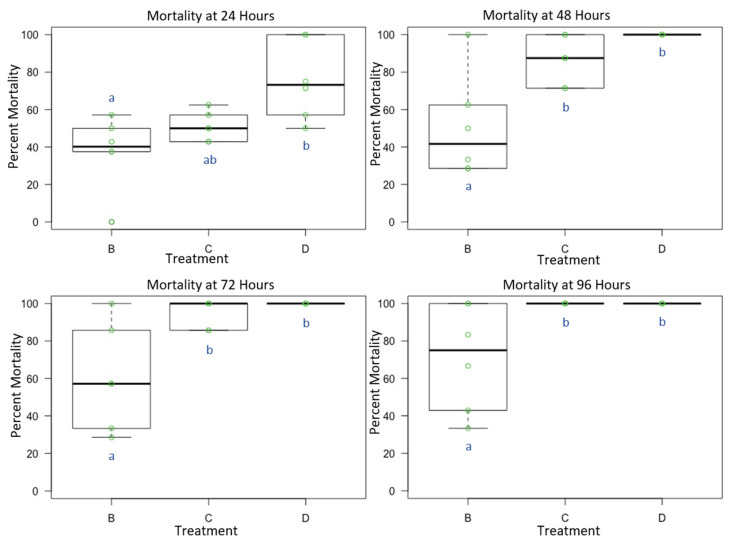
Female *Osmia lignaria* Acute Contact Toxicity Following Whole-Body Contact Exposure to Individual Active Ingredient Insecticides and their 1:1 Binary Combination: Female *Osmia lignaria* mean, 1st and 3rd quartile percent mortality following whole-body contact exposure to each individual active ingredient insecticide (Experiment 3) at 24 and 48 h. Data on the x-axis indicate each insecticide treatment, and data on the y-axis indicate percent mortality. Insecticide treatments: B = I, C = BC, D = IBC. All jars in a treatment containing the respective percent mortality are represented by a single green point. Significant difference at *p* < 0.05 (pairwise comparison) is indicated by different letters (a, b). Information related to control is given in the result section.

Female *O. lignaria* whole-body contact exposure to the individual active ingredient insecticides and their 1:1 binary combination resulted in statistically significant mortality between treatments at 24 h (F = 6.3879, *p* < 0.05, df = 2), 48 h (F = 11.266, *p* < 0.05, df = 2), 72 h (F = 10.972, *p* < 0.05, df = 2) and 96 h (F = 8.2398, *p* < 0.05, df = 2) (Figure 4). At 24 h, we observed that the 1:1 binary combination (IBC) was more toxic than I (*p* < 0.05). Similarly, at 48, 72 and 96 h, I was also less toxic than BC and the 1:1 binary combination (IBC) (*p* < 0.05). At 24 h, average control mortality was 6.25% with a standard error of 2.8%, while at 48 h, average control mortality was 10.42% with a standard error of 3.84%. Cumulative control mortality during the extended observational period at 72 h was averaged 14.58% with a standard error of 2.08%, and at 96 h, it was 20.83% with a standard error of 2.64%.

## 4. Discussion

In this study, direct whole-body contact exposure of *O. lignaria* to the formulated premix insecticides TLC and IBC resulted in approximately 40% mortality at 24 h. Mortality increased over time, and at 48 h, we observed approximately 80% mortality for both treatments, while complete mortality resulted for both treatments at 72 h. Although we removed control mortality from our data analysis by using Schneider-Orelli’s formula (Equation (1)), the relatively low control mortality at 48 h enabled us to demonstrate that *O. lignaria* mortality at 24 and 48 h increases as a result of exposure to neonicotinoid + pyrethroid combinations. Conversely, the increased control mortality at the extended screening observations complicates our conclusion of increased treatment mortality resulting from exposure to neonicotinoid + pyrethroid combinations during this extended period. However, as discussed in Phan et al. [19] standard EPA mortality readings that only extend 48 h post-exposure are not adequate for measuring the delayed mortality in *Osmia* spp. Therefore, our study builds upon this fallacy by improving the experimental design for pesticide mortality screening studies in *Osmia* and other non-*Apis* bees. Nonetheless, we observed quick knockdown characterized by trembling and an incapacity to fly within 25 min of application of these two treatments. Both male and female *O. lignaria* displayed the same behavior at 24 and 48 h. We primarily attribute this rapid knockdown to the presence of pyrethroid active ingredients, given their characteristic knockdown mode of action as sodium channel modulators (Table 1; [20]). Neonicotinoids have systemic and translaminar properties [21], which facilitate their movement into leaf tissue, thereby reducing bee contact to these chemicals after residues dry. Standard EPA LD_50_ values of these active ingredient chemistries have been quantified for *A. mellifera* [22,23,24,25,26]. Although *A. mellifera* cannot be considered as a surrogate for non-*Apis* bees including *O. lignaria* [27], these standard LD_50_ values provide a preliminary indication of the high toxicity of these insecticides to bees. It would be helpful to generate standard EPA LD_50_ values for insecticide toxicity to *Osmia* spp. bees for accurate comparison in future studies [19]. In this study, higher mortality could also be due to additive toxicity arising from exposure to both active ingredients of formulated pesticide mixtures, however, future studies with dose-mortality curves are necessary to examine any such effects [28]. Nonetheless, it is evident that *O. lignaria* will likely experience rapid mortality following whole-body contact exposure to TLC and IBC. It is interesting to note that females experienced higher mortality than males at 24 h, given their larger body size and increased bodily hair. Therefore, future studies analyzing physiological and metabolic differences between adult *O. lignaria* sexes are warranted.

Initially, reduced mortality was observed for CLC and MS. However, at 96 h, these treatments resulted in almost complete mortality. We observed some knockdown effect (characterized by inability to fly) for CLC at 24 h. As before, we attribute this finding to the pyrethroid active ingredient given its characteristic knockdown mode of action as a sodium channel modulator (Table 1; [20]). However, this knockdown was less pronounced than that for TLC and IBC, which we attribute to the lack of a neonicotinoid active ingredient. Moreover, the delayed toxicity of CLC to male *O. lignaria* is evident by our only recording complete mortality at 96 h. Interestingly, we recorded complete mortality (with one outlier) for females at 72 h, which is surprising given their physiological differences (as discussed for neonicotinoid + pyrethroid premix insecticides). As previously mentioned, the standard EPA LD_50_ values of these active ingredient chemistries to *A. mellifera* [24,25,26,29,30,31] are not a direct comparison because *A. mellifera* can differ in response to toxicants compared to non-*Apis* bees [27]. However, these standard EPA LD_50_ values are a starting point and do indicate lower toxicity of these insecticides to bees. Interestingly, Smagghe et al. [32] found acute and chronic toxicity of chlorantraniliprole to *Bombus terrestris* [(Hymenoptera: Apidae) (Linnaeus)] (a non-*Apis* bee) following oral consumption. This finding combined with ours supports the need for more research regarding the risk posed to non-*Apis* bees by chlorantraniliprole, which is a ryanodine receptor agonist (Table 1; [33]). Conversely, we observed the least amount of mortality for male *O. lignaria* treated with MS (and no knockdown activity), which we primarily attribute to the lack of pyrethroid chemistry and the lack of methoxyfenozide toxicity to bees. As with CLC, our recording of approximately 65% male mortality at 96 h indicates the slow onset of toxicity for MS. Similarly, it is surprising that we observed a slow kill for females exposed to MS, that was only apparent at 96 h. This finding contradicts the low EPA LD_50_ values for technical grade spinosad (insecticide class of spinetoram) acute contact toxicity to adult *A. mellifera* workers of 0.0029 µg formulation/bee [34]. Similarly, toxicity of spinosad formulated product residues on foliage to *A. mellifera* was quantified as inducing 25% mortality in caged bees exposed to field-realistic spray applications less than three hours after exposure [34]. Therefore, future research directly comparing differences in acute contact toxicity of spinosad chemistries to *Osmia* spp. versus *A. mellifera* (*Apis* versus non-*Apis* bees) is warranted.

For males, these results are not entirely surprising given the classification of methoxyfenozide as a diacylhydrazine ecdysone receptor agonist (Table 1; [33]) designed to induce premature molting in immature Lepidoptera [35]. Studies demonstrating the benign toxicity of methoxyfenozide [36] and reduced toxicity of spinetoram [37] to *B. terrestris* (a non-*Apis* bee) further support our results. Additionally, the translaminar movement of spinetoram [38] into leaf tissue reduces bee contact after residues have dried. These results demonstrate that male *O. lignaria* will likely experience delayed mortality following whole-body contact exposure to CLC and MS.

Female *O. lignaria* sprayed with CLC and MS experienced a slower onset of initial mortality, and specifically for MS. Interestingly, for females, CLC resulting mortality was close to that of TLC and IBC, which is a trend we did not observe for males. Moreover, compared to males, we observed higher mortality for females exposed to CLC at 24 and 48 h, and for females exposed to MS at 72 and 96 h. As with TLC and IBC at 24 h, this is surprising given the physiological differences between males and females. Therefore, as previously mentioned, future studies should investigate these differences. Female *O. lignaria* treated with CLC also displayed some knockdown behavioral effects (characterized by inability to fly) at 24 h. As before, we attribute this finding to the pyrethroid active ingredient. However, this knockdown was less pronounced than that for the TLC and IBC treatments which we attribute to the lack of a neonicotinoid. Conversely, we observed no knockdown activity for females treated with MS, which we primarily attribute to the lack of a pyrethroid. Our results for females agree with the findings of Smagghe et al. [32] demonstrating the risk of chlorantraniliprole to a non-*Apis* bee. Similarly, they are in agreement with the findings of Mommaerts et al. [36] showing the benign toxicity of methoxyfenozide and Besard et al. [37] depicting the reduced toxicity of spinetoram to a non-*Apis* bee. Such differences in results (and particularly for male versus female *O. lignaria*) warrant further assessment of metabolic differences in insecticide detoxification over an extended period. Given the rapid versus slow onset of mortality in this study, it would be helpful to explore *O. lignaria* detoxification at 24 h for highly toxic insecticides (TLC and IBC) and at 72 and 96 h for less toxic insecticides (CLC and MS), as has been done for insecticide combinations on *A. mellifera* [39]. In summary, our findings indicate that neonicotinoid + pyrethroid premix insecticides are highly toxic to both male and female *O. lignaria* as evidenced by the rapid onset of mortality observed at 24 and 48 h post treatment. Conversely, our findings indicate that premix insecticides comprised of different chemistries (diamide, ecdysone receptor agonist and spinosad) are less toxic to *O. lignaria* as evidenced by the slower onset of mortality that was primarily observed at 72 and 96 h post-treatment.

Under the scenario simulating whole-body contact exposure to individual active ingredients and their 1:1 binary combination, we found high mortality for male and female *O. lignaria* in all treatments, and specifically for the bees treated with the 1:1 binary combination. At 24 h for all treatments, we noticed that all alive male bees tremored, characterized by their wings, legs and antennae slightly moving. These behaviors are consistent with the mode of action for pyrethroid active ingredients as in Vijverberg et al. (Table 1; [20]) given their characteristic knockdown mode of action as a sodium channel modulator. Bees exposed to the I and BC treatments experienced similarly high levels of mortality at the 96 h screening. This finding suggests that the IBC 1:1 binary combination may result in a higher speed of kill, but not necessarily increased toxicity. Future work repeating these bioassays that aims to elucidate this uncertainty is therefore warranted. Additionally, our laboratory synthesized 1:1 binary combination was not an identical simulation of Leverage 360^®^ (the premix insecticide comprised of these two active ingredients), which contains 0.24 g/mL imidacloprid and 0.12 g/mL beta-cyfluthrin [40]. Additionally, the higher toxicity observed in this experiment is likely explained by the higher percentage of active ingredients in the single active ingredient products (Table 4) compared to those in the premix insecticide products (Table 2). Nonetheless, our results demonstrate that *O. lignaria* coming into whole-body contact with I, BC and particularly IBC will be unlikely to survive. However, future assessment of single active ingredient insecticides containing equal quantities of active ingredients as the premix insecticides we tested would be helpful in specifically determining the risk posed by exposure to insecticide mixtures to *O. lignaria*.

High control mortality may have resulted from several variables in our experimental design. First, the experimental jars that blue orchard bees were placed in the following treatment is an artificial environmental setting that does not ideally simulate *O. lignaria* foraging between their nest and an open field or orchard. Moreover, placing the experimental jars with treated bees within incubators set to (21 ± 0.5 °C; 65 ± 5% RH) and 16 h daylight and 8 h darkness is not completely representative of field realistic conditions. Additionally, feeding treated bees 50% sucrose does not accurately represent the floral nectar and pollen resources that managed *Osmia* spp. bees would feed on in a field or orchard. Therefore, this artificial environment may have stressed the bees and therefore contributed to their high mortality. Second, *O. lignaria* cocoons were stored in an incubator setting for three to seven weeks prior to use in experiments. Therefore, it is possible that this prolonged storage period utilized their lipid reserves and as a result, affected the fitness of the eclosed adult bees as discussed in James and Pitts-Singer [41]. Third, although *O. lignaria* are a spring bee that in the wild eclose in early spring, we ran our experiments in the summer months. Specifically, we ran Experiments 1 and 2 in June and Experiment 3 in July, and stimulated bees to eclose during this time frame by setting the incubator temperature. Therefore, it is possible that this temporal difference in the adult life stage impacted adult bee fitness. Fourth, a radiator behind the incubator we originally placed cocoons into overheated, resulting in many bees emerging prematurely. While none of the bees used in our experiments emerged prematurely, they did originate from the same cohort of cocoons that did. As a result, it is possible that the physiology of the bees we used for experiments may have been altered as a result of being exposed to the overheated environment.

Premixed insecticides containing pyrethroids are currently used for control of various orchard pests. However, they are generally not recommended by Integrated Pest Management (IPM) practitioners because of their disruptive effects on the biological control of secondary pests such as mites, aphids and scale insects. Nonetheless, several studies have validated their effective properties. For instance, the premix formulation containing TLC resulted in 66.7% brown marmorated stink bug [*Halyomorpha halys* (Hemiptera: Pentatomidae) (Stål)] mortality in 2011, and 80% mortality in 2012 [42]. In another study, Tanigoshi et al. [43] found high efficacy of TLC and IBC for spotted wing drosophila (SWD) [*Drosophila suzukii* (Diptera: Drosophilidae) (Matsumura)]. Similarly, Tanigoshi et al. [44] found IBC exceeded 90% SWD mortality, while TLC provided 89.7% efficacy. Insecticide control of oblique banded leaf-roller [*Choristoneura rosaceana* (Harris) (Lepidoptera: Tortricidae)] includes chlorantraniliprole, methoxyfenozide and spinetoram alone [45]. Premixes are primarily used to combat resistant leafroller populations, although alternating insecticides of differing modes of action is generally recommended as a better practice by IPM experts [46].

*O. lignaria* pollinate different orchard crops [2,4]. Application of the premix insecticides we tested in orchards is only permitted before bloom or after petal fall [40]. Spraying before bloom does not guarantee pollinator safety because systemic and translaminar insecticides including neonicotinoids [21] and spinetoram [38] may remain in plant tissues [47,48] and subsequently move from leaves and stems to pollen and nectar [49]. Dried insecticide residues applied before bloom may liquify in plant guttation fluids which can contain high neonicotinoid levels [50,51]. Bees have been found to collect guttation fluid [52], thereby potentially exposing them to insecticides. Our whole-body contact experiments placing anesthetized bees in Petri dishes into the spray tower where they were directly sprayed aims to simulate these combined scenarios. However, it is not a perfect replication of all potential insecticide exposure routes mentioned. Therefore, future research that separately simulates each of these scenarios more accurately than an overall combination like we carried out here is warranted. Applying insecticides after petal fall is a variable definition because flowering occurs in different stages due to temperature fluctuations [53]. Therefore, *O. lignaria* may forage flowers that bloom later in an orchard coinciding with when insecticides are applied. We designed our whole-body contact exposure bioassays to additionally simulate this scenario.

*Osmia* spp. bees also have been shown to be effective pollinators of berry crops [54,55,56]. Given that insecticide applications are not prohibited during the bloom of berry crops (for example, EPA [40]) *Osmia* species that are used to pollinate berries are at risk for insecticide exposure. Moreover, since bloom in berries can occur multiple times throughout a growing season in a fluctuating pattern [57], it is possible for *Osmia* spp. foraging berry agro-ecosystems to come into contact with insecticides as we simulated in our laboratory bioassays with *O. lignaria*. Additionally, field crop scenarios where sprays are made during bloom, while bees are simultaneously actively pollinating should be further examined. Examples would be cucurbit crops as discussed in Dively et al. [58] and canola as discussed in Sharma et al. [59] where blooms last several weeks and coincides with insect pests needing to be controlled at the same time. While these cited studies are primarily focused on *A. mellifera*, future work should investigate these scenarios on *Osmia* spp. bees. Testing the acute contact toxicity of the insecticides we used in this study at field-realistic rates for berry crops to *O. lignaria* should be examined in future studies.

The toxicity of insecticides to commercially managed *Osmia* spp. has been demonstrated in a few studies. In *O. bicornis*, a negative correlation between egg cell production and neonicotinoid residues [60] and decreased reproduction, increased mortality and male-biased sex ratios after thiamethoxam oral consumption [61] were found. In *O. cornifrons*, contact exposure to formulated lambda-cyhalothrin dissolved in water demonstrated significantly higher toxicity compared to imidacloprid, acetamiprid and phosmet at LD_50_ [27], while *O. lignaria* larval consumption of technical grade imidacloprid dissolved in water led to prolonged development and cocoon spinning times [7]. Placing *O. bicornis* adjacent to clothianidin + beta-cyfluthrin seed-coated fields decreased nest construction and provisioning [62]. Conversely, in a similar study exposing *O. bicornis* to clothianidin + beta-cyfluthrin, Peters et al. [63] concluded no negative impacts determined by high reproduction and low parasitism levels.

As previously mentioned, the premix insecticides we tested are widely used in agricultural pest control. However, to our knowledge, no study of whole-body acute contact toxicity of these premix insecticides to *O. lignaria* has been published. Future work that incorporates both technical grade products dissolved in acetone and formulated products dissolved in water is necessary for accurate assessment of insecticide risk to *O. lignaria* and other non-*Apis* species (especially given the contrasting results in these current studies).

## 5. Conclusions

The results of this study demonstrate the differences in toxicity (as measured by the onset of mortality) of formulated premix insecticides and their individual active ingredients to *O. lignaria*, which are increasingly being used for pollination. However, a direct assessment of toxicity of technical grade versus formulated products that assess multiple doses to generate LD_50_ values is required. Given our finding of (1) differences in male versus female mortality and (2) differences in the timing of premix insecticide mortality onset (24–48 h for neonicotinoids + pyrethroids, and 72–96 h for diamides, ecdysone receptor agonists and spinosads), examining this over an extended period is crucial. Modification of regulatory protocols to assess insecticide toxicity to non-*Apis* bees over extended periods will be supported by these data [19,27].

Future studies should assess whether additive or synergistic toxicities exist between these premixed insecticides to *O. lignaria* by testing multiple doses to generate dose-mortality curves and test the hypothesis of parallel versus equal slopes. It would be interesting to explore whether *O. lignaria* molecular-level insecticide detoxification is impaired as a result of being exposed to the premixes containing multiple active ingredients (each with a different mode of action). Finally, future work should analyze sub-lethal and chronic insecticide toxicity to *O. lignaria*. Specifically, it would be interesting to observe if exposure to lower concentrations of the premix insecticides we tested negatively impairs the normal behaviors of this bee species. It is critical to determine if contact exposure to these insecticides impairs male and female blue orchard bee collection of pollen and nectar, male fertilization of females, female nest provisioning and egg oviposition, and proper development of immature brood into adult bees.

## Figures and Tables

**Table 1:** Active ingredients and modes of action of formulated premix insecticides assessed for acute whole-body contact toxicity to adult male (Experiment 1) and female (Experiment 2) *Osmia lignaria*.

Treatment	Formulation	Active Ingredient(s)	Mode of Action 1st Insecticide	Mode of Action 2nd Insecticide	Manufacturer
A	Control (distilled water)	-	-	-	-
B	Endigo ZC^®^	Thiamethoxam (12.6%) + Lambda-cyhalothrin (9.48%)	Thiamethoxam -Nicotinic acetylcholine receptor (nAChR) competitive modulator	Lambda-cyhalothrin -Sodium channel modulator	Syngenta Crop Protection, LLC Greensboro, NC
C	Leverage 360^®^	Imidacloprid (21.0%) + Beta cyfluthrin (10.5%)	Imidacloprid -Nicotinic acetylcholine receptor (nAChR) competitive modulator	Beta-cyfluthrin -Sodium channel modulator	BayerCropScience LP Research Triangle Park, NC
D	Besiege^®^	Chlorantraniliprole (9.26%) + Lambda-cyhalothrin (4.63%)	Chlorantraniliprole -Ryanodine receptor modulator	Lambda-cyhalothrin -Sodium channel modulator	Syngenta Crop Protection, LLC Greensboro, NC
E	Intrepid Edge^®^	Methoxyfenozide (28.3%) + Spinetoram (5.66%)	Methoxyfenozide -Ecdysone receptor agonist (molt accelerating compound)	Spinetoram -Nicotinic acetylcholine receptor (nAChR) allosteric modulator	Dow AgroSciences LLC Indianapolis, IN

**Table 2:** Application concentration administered by spray application, highest recommended application concentration, and active ingredient concentrations of formulated premix insecticides assessed for acute whole-body contact toxicity to adult male (Experiment 1) and female (Experiment 2) *Osmia lignaria*.

Treatment	Active Ingredient(s)	Application Concentration Administered (mL/Ha)	Highest Application Concentration on Label (mL/Ha) *	AI Concentration (ppm) in Formulated Product (per L)
A	Control (distilled water)	-	-	-
B	Thiamethoxam (12.6%) + Lambda-cyhalothrin (9.48%)	401.76	438.28	Thiamethoxam = 120 Lambda-cyhalothrin = 91
C	Imidacloprid (21.0%) + Beta cyfluthrin (10.5%)	189.92	204.53	Imidacloprid = 98 Beta-cyfluthrin = 49
D	Chlorantraniliprole (9.26%) + Lambda-cyhalothrin (4.63%)	657.42	876.56	Chlorantraniliprole = 56 Lambda-cyhalothrin = 28
E	Methoxyfenozide (28.3%) + Spinetoram (5.66%)	657.42	876.56	Methoxyfenozide = 140 Spinetoram = 28

* Highest recommended application rate for orchard crops listed on the Environmental Protection Agency regulated product label for each respective insecticide.

**Table 3:** Active ingredients and modes of action of individual active ingredient insecticide assessed for acute whole-body contact toxicity to adult male and female *Osmia lignaria* (Experiment 3).

Treatment	Formulation	Active Ingredient	Insecticide Mode of Action	Manufacturer
A	Control (distilled water)	-	-	-
B	Admire Pro^®^	Imidacloprid (42.8%)	Imidacloprid -Nicotinic acetylcholine receptor (nAChR) competitive modulator	BayerCropScience LP Research Triangle Park, NC
C	Baythroid XL^®^	Beta-cyfluthrin (12.7%)	Beta-cyfluthrin -Sodium channel modulator	BayerCropScience LP Research Triangle Park, NC
D	(1:1 binary combination by volume) of Treatment B and Treatment C	Imidacloprid + Beta-cyfluthrin	-	-

**Table 4:** Application concentrations administered, highest recommended application concentrations, and active ingredient concentrations of individual active ingredient insecticides assessed for acute whole-body contact toxicity to adult male and female *Osmia lignaria* (Experiment 3).

Treatment	Active Ingredient	Application Concentration Administered (mL/Ha) *	Highest Application Concentration on Label (mL/Ha) *	AI Concentration (ppm) in Formulated Product (per L)
A	Control (distilled water)	-	-	-
B	Imidacloprid (42.8%)	102.27	204.53	Imidacloprid = 120
C	Beta-cyfluthrin (12.7%)	102.27	204.53	Beta-cyfluthrin = 26
D **	Imidacloprid + Beta-cyfluthrin	-	-	-

* Highest recommended application rate for orchard crops listed on the Environmental Protection Agency regulated product label for each respective insecticide. ** Proportion of active ingredients in 1:1 binary mixture made in 100 mL water solvent (Imidacloprid—2 mL and Beta-cyfluthrin—1 mL).

## Data Availability

Data from this study is available from the corresponding author on reasonable request.
